# The Impermanence of Public Health Gains—Lessons Learned from Measles Resurgence, Bangladesh, 2026

**DOI:** 10.3201/eid3211.261019

**Published:** 2026-11

**Authors:** Saju Bhuiya, Tanzir Ahmed Shuvo, Md Zakiul Hassan, Mohammad Abdul Aleem, Gazi Md Salahuddin Mamun, A.F.M. Zakaria, Mahmudur Rahman, Fahmida Chowdhury

**Affiliations:** icddr,b, Dhaka, Bangladesh (S. Bhuiya, T.A. Shuvo, M.Z. Hassan, M.A. Aleem, G.M.S. Mamun, F. Chowdhury); Shahjalal University of Science and Technology, Sylhet, Bangladesh (A.F.M. Zakaria); The Eastern Mediterranean Public Health Network, Dhaka (M. Rahman)

**Keywords:** Measles, measles resurgence, viruses, respiratory infections, immunization coverage, health system resilience, Bangladesh

## Abstract

The global resurgence of measles is a major public health concern. After the success of its Expanded Programme on Immunization, Bangladesh experienced a measles resurgence in 2026. The country faced major setbacks after measles vaccination coverage declined because of political instability, discontent among field-level staff, change in procurement policy, COVID-19–related service disruptions, and delayed supplementary immunization campaigns. During March 15–September 13, 2026, Bangladesh reported 170,638 suspected and 20,006 confirmed measles cases, including 919 deaths among suspected and 100 deaths among laboratory-confirmed cases. The outbreak disproportionately affected unvaccinated and underimmunized children. Political turmoil during 2024–2025 disrupted vaccine procurement, delayed mass vaccination campaigns, weakened the health workforce, and compromised financial and logistic continuity. Simultaneously, disruptions in vitamin A supplementation and worsening child malnutrition amplified disease severity. To prevent future resurgence, immunization programs must be depoliticized, supported by buffer vaccine stocks, strengthened through microlevel surveillance, and integrated with nutrition and primary healthcare services.

Measles is an extremely contagious viral disease; each primary case generates an average of 12–18 secondary infections (*1*). Although global measles cases declined by 71% from 2000 to 2024, the World Health Organization (WHO) reported 395,521 cases in 2024 and 254,384 in 2025; by the end of April 2026, global case counts had reached 86,503 (*1*,*2*). Like other countries, Bangladesh’s measles outbreak during early 2026 served as a clear reminder that public health achievements are never permanent; they are dynamic states of equilibrium that require constant maintenance, political or structural stability, and resource security. 

Bangladesh was praised as a global exemplar of the Expanded Programme on Immunization (EPI) for decades; its successful vaccination coverage increased from 2% in the late 1970s to near-universal levels by the 2010s (*3*,*4*). Measles-containing vaccine (MCV) 1 coverage increased from 74% in 2000 to 94% in 2016, and MCV2, a routine second MCV dose introduced in 2012, reached 93% in 2016 (*5*). Bangladesh achieved its regional rubella control goal in 2018 and subsequently targeted measles and rubella elimination by 2020 (*6*). However, the COVID-19 pandemic and the early 2026 measles outbreak have challenged that narrative, revealing underlying weaknesses in Bangladesh’s health system. The 2026 measles outbreak was not merely a biological phenomenon driven by a highly contagious virus, it was a systems-sensitive emergency that exposed the intersection of vaccination disruption resulting from political upheaval (Appendix Table), nutritional neglect, disruption of regular vaccine procurement, perceived adverse effects of COVID-19 vaccination, widespread misconceptions, and the long-term disruption of routine immunization services in the aftermath of the COVID-19 pandemic (*7*–*9*). 

Previous publications have documented the early measles resurgence, immunization gaps, vaccination disruptions, and health-system pressures in Bangladesh (*7*,*10*–*12*). Building on the evidence in those publications, this perspective provides an integrated analysis of the epidemiologic, demographic, nutritional, socioeconomic, political, and health-system factors that might have contributed to the magnitude and severity of the outbreak, while drawing lessons for strengthening measles prevention, preparedness, and response in Bangladesh and similar settings.

## Summary of the 2026 Measles Outbreak in Bangladesh

The measles outbreak came to the attention of the government and other health agencies on March 15, 2026, and by September 13, 2026, Bangladesh reported 170,638 suspected and 20,006 confirmed cases and reported 919 deaths among suspected and 100 deaths among laboratory-confirmed cases (*13*). Case-fatality rates were 0.54% among suspected and 0.50% among confirmed cases (*13*). Case definitions were described previously (*14*). 

Genomic analysis of 55 measles virus whole-genome sequences showed that all virus samples belonged to the B3 subclade (*15*). Phylogenetically, the Bangladesh viruses were closely related to B3 viruses reported from other countries (including Bangladesh), supporting circulation of a globally recognized lineage rather than emergence of a distinct vaccine-escape strain (*16*,*17*). Four amino acid changes were identified within antigenic regions of the haemagglutinin protein; however, several of those changes were previously reported in measles viruses from other settings (*15*). Those amino acid changes alone do not establish reduced vaccine susceptibility, and further immunologic and functional studies are required to determine their potential effect on antibody recognition. Overall, the genomic findings provided no evidence of a novel measles lineage or genetic evolution sufficient to explain the current resurgence through vaccine escape. 

The 2026 measles outbreak in Bangladesh is characterized by its clinical severity and its extensive geographic spread; all 64 districts in the country are affected (*18*). By May 3, 2026, the Directorate General of Health Services (DGHS) and WHO identified Bangladesh’s capital city, Dhaka, as the primary epicenter, and noted that cases were concentrated in overcrowded informal settlements (*13*,*18*). The spatial concentration of the 2026 outbreak in Dhaka’s informal settlements (slums) highlights gaps in urban health governance (*19*).

During this outbreak, hospitals, particularly the Infectious Diseases Hospital (IDH) in Mohakhali, Dhaka, were overwhelmed by a concentration of hypoxic, malnourished, and severely ill children, and in March 2026 the crude in-hospital mortality rate was 4.7% (*20*). That high mortality rate, concentrated in tertiary referral hospitals, suggests potential gaps in identifying and treating measles in secondary and primary healthcare centers. Moreover, our data only reflect hospitalized patients, and the burden among persons who did not seek healthcare at the community level remains unknown (*14*).

## Potential Factors Contributing to the Bangladesh Measles Outbreak

### Political Upheaval and the Disruption of Routine Immunization Activity, 2024–2025

Bangladesh’s routine immunization program provided 2 doses of measles–rubella vaccine free of charge through the EPI. The first dose is administered to infants at 9 months of age and the second at 15 months (*5*). Vaccination is available at all government hospitals, upazila (subdistrict) health complexes, dedicated public clinics, and participating private maternal centers. However, the political instability in Bangladesh during 2024 and 2025 (Appendix Table) disrupted routine immunization and was associated with the 2026 measles outbreak.

A student-led uprising in 2024 and the subsequent political transition from the prior administration disrupted administrative continuity and had a consequential effect on public health administrative and leadership during the interim caretaker government’s transitional rule (*3*,*4*,*10*). That period of institutional reorganization unintentionally disrupted routine decision-making channels and interrupted operational continuity within the Ministry of Health and Family Welfare (MoHFW). Consequently, systematic EPI oversight was disrupted, delaying scheduled nationwide vaccination campaigns. Before administrative stability was fully restored, the cumulative oversight gaps allowed the cohort of immunologically naive children to expand, ultimately transforming a localized coverage deficit into a widespread nationwide outbreak.

For years, Bangladesh procured vaccines through UNICEF (https://www.unicef.org) with major support from Gavi (https://www.gavi.org) and government cofinancing. After the 2024 political transition, the interim government shifted to an open tender system in 2025, causing procurement delays, nationwide vaccine shortages, disruption in routine immunization, and cancellation of the measles vaccine campaign (*21*). That shift in measles vaccine procurement from UNICEF to open tender system contributed to nationwide vaccine stockouts. Those stockouts, in turn, extended the population immunity gap by expanding the cohort of immunologically naive children 6–9 months of age, thereby fueling the rapid resurgence of measles (*21*).

Nevertheless, the newly appointed Health Minister, Sardar Mohammed Sakhawat Husain, reportedly told in the Jatiya Sangsad (Bangladesh’s national parliament) that the political turmoil over the previous 2 years had disrupted vaccine procurement and caused failures in routine national vaccination campaigns (*4*). Thus, the 2026 outbreak might not have been a failure of medical technology but rather a failure of the health system function, amplified by prolonged disruptions in immunization services and program oversight.

### Accumulation of Unvaccinated Children and Disrupted Follow-up Immunization Campaigns

One of the primary drivers of the 2026 outbreak was the accumulation of susceptible children. The follow-up measles and rubella vaccination campaign, a door-to-door campaign generally conducted every 4 years as a catch-up strategy, is intended to reach children missed by fixed-site services, particularly those in hard-to-reach, mobile, poor, and urban-slum populations (*10*). The last follow-up campaign took place in 2020; the next was scheduled for 2024 (*3*,*11*). However, the political instability in 2024, combined with widespread health worker strikes and funding uncertainties, led to the indefinite postponement of that critical immunization safety net (*3*,*10*,*11*,*22*). That administrative failure resulted in a substantial cohort of zero-dose (i.e., unvaccinated) children, creating a critical immunity gap within the pediatric population (*10*). UNICEF assessments during the outbreak found that 72% of cases were among zero-dose children, and another 16% were among partially vaccinated children (*11*). Another report showed that by 2025, Bangladesh had ≈5 million children who were not fully immunized, including 70,000 who had received no doses and >400,000 who were underimmunized (*20*). That demographic vulnerability resulted directly from disruptions in routine immunization and the failure to implement catch-up mechanisms that could identify and reach unimmunized children.

In addition, neither national nor international organizations highlighted that critical immunization gap on any platform or in the media, and no red flag was raised by the EPI before 2026. That institutional silence stemmed from a combination of data-tracking failures and bureaucratic distractions after the 2024 political transition. Specifically, routine EPI surveillance relied on underestimated population denominators, which obscured the true magnitude of the decline in vaccination coverage (*23*).

Furthermore, the surveillance system lacked automated risk-linked thresholds to trigger early warnings. The transition from UNICEF prefinancing to an open tender procurement system diverted leadership attention toward logistic restructuring rather than epidemiologic risk assessment. Consequently, international agencies favored private diplomatic dialogue over public reporting. Those organizations might have exercised diplomatic caution and avoided public platform alerts to maintain working relationships with the sensitive postrevolutionary interim administration, inadvertently keeping the gap out of public and media view and leaving the widening immunologic vulnerability unaddressed until the 2026 outbreak.

### Longitudinal Erosion of Vaccine Coverage during 2014–2024

The 2026 crisis was associated with a documented, multiyear decline in vaccination coverage that exacerbated the outbreak. Although crude national figures often remained above the 90% vaccine threshold, a closer analysis of valid coverage vaccines administered at the correct age and interval revealed a more precarious immunologic reality (*24*). National coverage metrics use aggregate data that frequently mask localized pockets of underimmunization, an epidemiologic vulnerability known as the fallacy of the average (*25*). When unvaccinated populations cluster geographically, such as in densely populated urban slums or remote rural subdistricts in hard-to-reach areas, effective local herd immunity falls far below the critical threshold required to prevent transmission (*25*). In Bangladesh, that structural error was compounded by denominator errors that omitted millions of children from official targets, allowing highly vulnerable, localized cohorts to expand unnoticed until they achieved the critical mass necessary to fuel an explosive nationwide resurgence of measles (*23*). Moreover, WHO immunization data suggest that the overall vaccination coverage decreased during and after the COVID-19 pandemic (*26*). Vaccination coverage further declined in 2025 (Figure), whereas concurrent adverse conditions contributed to progression toward the outbreak. 

**Figure:**
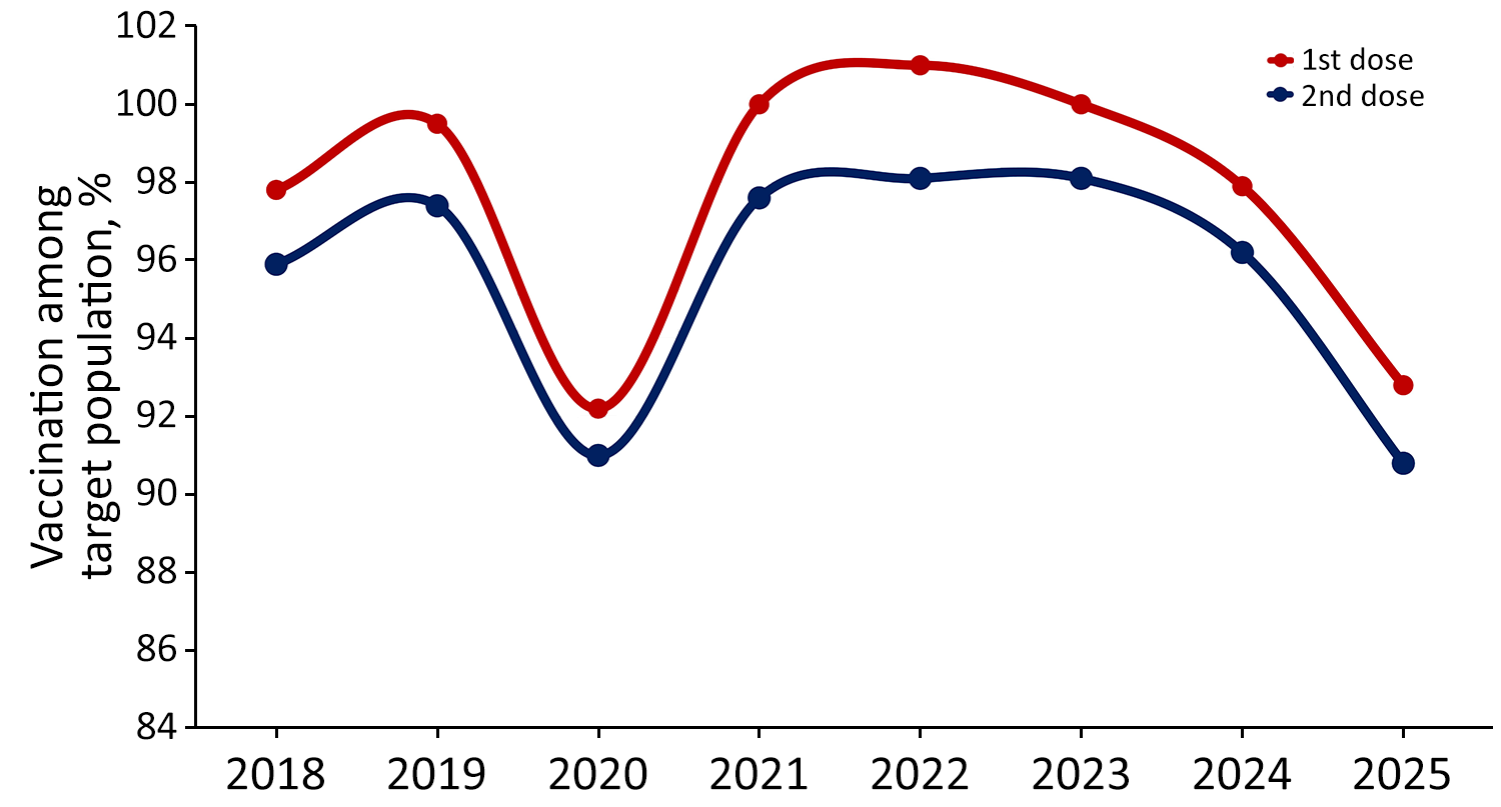
National measles-containing vaccination coverage trend among the eligible target population in study of lessons learned from measles resurgence, Bangladesh, 2026. Graph shows administrative vaccination data for children >9 months of age during 2018–2025. First measles-containing vaccine dose is recommended at 9 months of age and second dose at >15 months of age. Administrative data based on reported doses administered and estimated target population previously available in Directorate General of Health Services dashboard (https://dashboard.dghs.gov.bd).

Administrative data indicated a sharp decline in MCV coverage in 2020 (Figure). In addition, MCV1 coverage fell from 98% to 92% by 2025, while MCV2 coverage decreased from 96% to 91% in the same period (Figure). The 2023 Coverage Evaluation Survey reported full vaccination coverage of 86.1% nationally and that urban areas lagged behind rural regions (83.8% vs. 88.8%) (*24*). Although that difference was modest, aggregate estimates might mask pockets of lower coverage in densely populated urban slum areas. For example, the 2019 EPI Evaluation Survey reported only 75% valid vaccination coverage by 12 months of age among children in Dhaka slum areas, whereas overall Dhaka district coverage was 86.8%, illustrating how aggregate estimates can mask substantially lower coverage in specific urban populations (*25*,*27*). Such pockets of underimmunized populations enable rapid spread of measles in densely populated urban settings. In several countries, including Bangladesh, measles vaccination coverage decreased during and after the COVID-19 pandemic (*26*).

### Healthcare System Preparedness and Clinical Case Management

By March 2026, Bangladesh’s health system was under severe pressure, and by August 15, 2026, a total of 123,494 suspected measles patients were admitted to hospitals (*13*). The Infectious Diseases Hospital in Dhaka, originally a 100-bed facility, experienced severe overcrowding and limited intensive care capacity (*28*). The DGHS and WHO identified Dhaka as the primary outbreak epicenter, where a total of 62,286 suspected and 10,505 confirmed cases and 62 deaths had occurred by August 15, 2026; cases were mainly concentrated in overcrowded informal settlements (*13*,*18*). Moreover, another study showed that hospital bed occupancy generally exceeded 164%–175% in 2025, further limiting Bangladesh’s ability to manage seasonal surges (*29*). 

UNICEF and partners’ assessments identified several critical facility-level failures, including staffing shortages, nosocomial transmission, and logistical supply gaps (*11*,*28*). The government’s response, which included launching an emergency vaccination campaign for 1.2 million children in 18 high-burden districts on April 5, 2026, was a necessary tactical measure that highlighted the reactive nature of the health system (*11*,*18*). The outbreak also exposed critical systemic failures extending far beyond simple vaccination gaps, particularly in isolation capacity, infection prevention and control, and clinical case management. Despite DGHS and WHO directives for 4-day case isolation and extended precautions for immunocompromised patients, hospital overcrowding and weak referral systems drove nosocomial amplification and sustained transmission (*11*,*19*).

Furthermore, political instability in 2024 led to the reassignment of key personnel and persistent vacancies in senior EPI leadership positions (*30*), weakening program planning and implementation. Repeated healthcare worker strikes further disrupted routine immunization services (*31*).

### Legacy of COVID-19–Era Global and Regional Immunization Disruption 

The 2026 outbreak in Bangladesh is an extreme manifestation of the global resurgence of measles and the backsliding of immunization progress due to the COVID-19 pandemic (*8*,*9*). Moreover, 81% of cases were in children <5 years of age, highlighting the potential consequences of immunization disruptions during the COVID-19 pandemic (*13*). Worldwide data showed reduced measles vaccination during the pandemic (*8*,*26*). Pandemic-related disruptions to local immunization programs and rising vaccine hesitancy after the pandemic exacerbated this outbreak in Bangladesh (*32*). Similar results were reported in studies conducted in India and Pakistan (*8*,*33*,*34*). The measles outbreak in Bangladesh is a reminder that vaccine gaps do not simply disappear when a pandemic ends; rather, those gaps could accumulate.

## Drivers of High Rates of Illness and Death

The high rates of illness and death during the 2026 measles outbreak likely resulted from interacting epidemiologic, nutritional, socioeconomic, and health system vulnerabilities. Collectively, those vulnerabilities created a reinforcing pathway from high transmission and susceptibility to delayed care, severe complications, and excess mortality. We describe the vulnerabilities further in the following sections.

### Disruption in Vitamin A Supplementation

The severity of the 2026 measles cases was exacerbated by a parallel decline in nutritional interventions in Bangladesh. In 2025, disruptions to the national Vitamin A Plus campaign meant that only 1 of the 2 scheduled annual dosing rounds were completed (*11*). Vitamin A is crucial for reducing measles-related death; without it, children are more likely to experience severe complications (*33*,*35*). Hospitals also reported that the children admitted during the outbreak were malnourished and had weight-for-height percentages far below standard (*33*). According to 1 study, malnutrition acts as a powerful epidemiologic multiplier that accelerates measles transmission and severity (*36*). The MoHFW decision in April 2026 to prioritize available vitamin A stocks specifically for children with measles was a necessary but late-stage response to a problem that should have been addressed through preventive supplementation in 2025 (*11*).

### Demographic Vulnerability and Transmission Shift 

A deeply concerning trend observed during this outbreak was the high percentage of infections among infants <9 months of age (*19*). Historically, maternal antibodies protected infants against measles, but only through the first 6 months after birth, leaving many infants 6–9 months of age vulnerable to measles infection before they receive the first vaccine dose (*37*). As population immunity levels dropped below the 95% threshold required for herd protection, those unprotected cohorts became the primary victims of intense community transmission (*7*,*35*). In some hospitals, such as Rajshahi Medical College, up to 57% of confirmed cases involved infants <9 months of age (*38*). That pattern suggests that reduced vaccination coverage weakened the protective effect of population immunity, increasing infants’ exposure to measles virus. Moreover, hyperdense informal urban settlements formed localized micropockets of underimmunization, driven by high population mobility and a systemic deficit in routine surveillance tracking. 

In Dhaka, cases clustered heavily in informal settlements such as Demra, Kamrangirchar, and Korail slums (*19*). Those areas are characterized by high population density, limited visibility to health services, and environmental stressors (*7*,*19*,*20*,*33*). Dhaka division also reported the highest cumulative burden, with >62,286 suspected cases, highlighting how static immunization delivery models can fail to maintain herd immunity among transient populations during an outbreak and how current urban health infrastructure might be insufficient to meet the public health needs of a rapidly urbanizing population during a crisis (*13*,*18*,*19*). To resolve that operational mismatch, static healthcare delivery should be complemented by an agile, mobile vaccination framework. Flexible, microtargeted teams operating during nonstandard hours at transit hubs and markets could reach highly transient populations more effectively.

### Suboptimal Economic Condition

The 2026 measles surge imposed substantial economic hardship, and affected families spent an average of BDT 16,000 (Bangladeshi taka) on treatment and related costs, alongside income losses from prolonged caregiving (*39*). Among affected households, 89% borrowed money and 61% drew from their savings, highlighting the outbreak’s potential to cause economic loss and vulnerability (*39*). Those costs highlight the risk for catastrophic and impoverishing healthcare expenditure (*39*). The economic burden of measles also could worsen health outcomes by delaying care-seeking and limiting access to essential treatment, nutrition, and supportive care. 

## Strategies for Resilient Immunization Systems

The 2026 measles outbreak in Bangladesh is a tragic case-study in the failure of acceptable national averages. To prevent a recurrence, the following lessons could be integrated into future public health strategies.

### Tracking National Aggregate Coverage

Routine monitoring of national and subnational (district-level, urban, rural slum, and hard-to-reach areas) vaccination coverage is essential for identifying immunity gaps before they translate into outbreaks. Coverage data should be reviewed regularly according to age, geographic area, and relevant population characteristics to identify underimmunized populations. Timely identification of declining coverage would enable health authorities to target catch-up vaccination programs.

### Reducing Vaccine Misconceptions

Vaccine misconceptions could be reduced by disseminating accurate information through trustworthy media; professionals, such as physicians, vaccinologists, and teachers; and mass awareness campaigns (*29*,*32*). Moreover, a legal framework could help curb antivaccine activities and misinformation.

### Depoliticizing Public Health Logistics

The delay of the 2024 mass follow-up vaccination campaign because of political unrest was the single most avoidable cause of the 2026 surge in deaths (*3*,*4*). To address that delay, immunization programs must be treated as essential state functions with autonomous funding and procurement mechanisms protected from political transitions. Creating a national vaccine stock would provide a cushion during national crises (*7*,*11*).

### Integrating Syndemic Management

A syndemic approach requires addressing measles alongside the interacting health, social, and political conditions that amplify its impact (*40*). The 2026 crisis reflected a synergy between viral transmission and malnutrition, including vitamin A deficiency (*11*). Therefore, outbreak control should integrate vaccination with nutritional support, early identification and management of complications, and strengthened routine child health services. Follow-up vaccination should be incorporated into routine immunization activities rather than as an episodic emergency response (*7*,*20*).

### Developing Urban-Specific Immunization Strategies

Urban informal settlements can contain localized clusters of underimmunized children because of population mobility, overcrowding, socioeconomic disadvantage, and limited access to routine health services (*41*). We describe that pattern as the slum cluster phenomenon, referring to the concentration of susceptible or incompletely vaccinated children within densely populated informal settlements, which can enable sustained measles transmission after virus introduction (*41*,*42*). Immunization strategies in those settings should complement routine facility-based services through targeted outreach, mobile vaccination, and follow-up of children who have missed recommended doses (*41*). Urban areas like Dhaka require specialized mobile vaccination teams that operate according to the schedules and movement patterns of informal settlement residents, rather than administrative convenience (*7*,*41*).

### Linking Surveillance to Rapid Public Health Response

The 2026 outbreak was underway by December 2025, yet the emergency measles vaccination campaign was not launched until April 2026 (*3*,*11*,*18*), highlighting the need to link early warning with timely public health action. Routine surveillance of measles cases, hospital admissions, vaccination coverage, and other relevant indicators should be linked to predefined thresholds that trigger rapid epidemiologic assessment and, when warranted, activation of response teams and targeted vaccination activities (*19*,*20*). Other countries with similar healthcare settings also could adopt such initiatives.

## Conclusions

The 2026 measles resurgence in Bangladesh was not adequately explained by a single national coverage trend or by COVID-19 disruption alone. The outbreak was largely driven by accumulated immunity gaps resulting from disruptions in routine immunization, declining vaccination coverage, vaccine supply constraints, lack of health system preparedness, and missed supplementary immunization opportunities. Those vulnerabilities enabled sustained transmission, particularly among young and undervaccinated children, and were further amplified by population mobility and weaknesses in timely surveillance and response. Preventing future outbreaks requires sustained and equitable coverage of at least 95% with both measles-containing vaccine doses, reliable vaccine supply, and systematic identification and follow-up of missed-dose and zero-dose children. Maintaining adequate vaccine stocks, strengthening hospital infection prevention and clinical preparedness, and addressing vaccine misinformation should be integral to a resilient measles elimination program. 

In summary, the 2026 measles outbreak in Bangladesh demonstrates that strong historical performance does not ensure future protection; maintaining it requires constant vigilance, adaptive governance, and equitable healthcare delivery. The ≈803 lives lost in 2026 must serve as the impetus for a fundamental shift in how immunization is governed, funded, and delivered in an increasingly politically unstable world.

Appendix Additional information on lessons learned from measles resurgence, Bangladesh, 2026.
