# Supplementary material for: The Impermanence of Public Health Gains—Lessons Learned from Measles Resurgence, Bangladesh, 2026
Source: Emerg Infect Dis. 2026 Nov;32(11):1777–84. doi: 10.3201/eid3211.261019 (PMC13715886; doi:10.3201/eid3211.261019)
Supplement: Supplementary file 1 [file 26-1019-Techapp-s1.pdf]

Article DOI: https://doi.org/10.3201/eid3211.261019

EID cannot ensure accessibility for supplementary materials supplied by authors. Readers who have difficulty accessing supplementary content should contact the authors for assistance.

The Impermanence of Public Health Gains—Lessons Learned from Measles Resurgence, Bangladesh, 2026

Appendix

Appendix Table. Political disruption and its potential impact on health-system functioning and resurgence of measles outbreak in Bangladesh

Time frame	Political events	Immediate societal and health-system disruption	Potential relevance to immunization and outbreak preparedness
5-Jun-24	High Court reinstated a quota reserving 30% of civil-service posts for descendants of freedom fighters.	Triggered renewed student mobilization and political tension.	Marked the beginning of a period of escalating instability that subsequently affected routine public-sector activities.
6 June–5 July 2024	Student-led anti-quota protests intensified and expanded nationwide	Beginning with protests at Dhaka University on 6 June against the reinstatement of the government-job quota system, the movement expanded to universities across Bangladesh by 9 June. From 1–5 July, the Anti-Discrimination Student Movement organized nationwide rallies, sit-ins, road and railway blockades, and strikes.	These activities increasingly disrupted transport and population mobility, creating potential challenges for routine government operations, vaccination outreach, field supervision, and access to health facilities.
6–13 July 2024	Student protests expanded nationally, including blockades of major roads and railway lines.	Dhaka transport was severely disrupted; long-distance buses and trains were stopped, while highways and railway routes were blocked in several regions.	Disruption of population communication, routine visit in the household and field operations could impede vaccination outreach, supervision, and access to health facilities.
14–15 July 2024	Political rhetoric intensified and clashes between protesters and pro-government student groups escalated.	Violence spread to multiple universities and urban areas; healthcare facilities, including Dhaka Medical College Hospital, were affected during clashes.	Growing civil unrest created an increasingly unstable environment for any kind of routine activities perhaps routine public-health operations and healthcare delivery.
16-Jul-24	A student of Rangpur's Begum Rokeya University (Abu Sayed, age 25, a student of English Department; one of the organizers of the quota reform movement) was killed during a clash between police and protesters seeking quota reform on the campus.	Protests escalated into widespread violence following deaths and clashes in several locations. Road blockades expanded across Dhaka and other regions, while major highways and railway routes were obstructed. Schools and colleges were subsequently closed indefinitely.	Suspension of educational activities can disrupt school- and community-based vaccination activities and reduce opportunities to identify and reach missed children.
18 July–3 August 2024	Nationwide anti-quota protests escalated into widespread violence, a “complete shutdown,” curfew, and military deployment. The Supreme Court issued its quota verdict on 21 July. Although restrictions were	Schools and universities remained closed; public transport, railway and metro services were disrupted; road movement and public gatherings were restricted; nationwide internet and mobile communication were interrupted. Curfews and recurrent unrest disrupted	Movement restrictions and closure of educational institutions disrupted routine and school/community-based immunization, outreach, supervision, and identification of missed children. Interrupted communication channels also affected disease

Time frame	Political events	Immediate societal and health-system disruption	Potential relevance to immunization and outbreak preparedness
	gradually eased and communications restored from 24–28 July.	routine government and health-system operations, surveillance reporting, coordination, supervision, and access to health services.	surveillance, outbreak reporting, field coordination, and rapid dissemination of public-health information.
4-Aug-24	Widespread violence and nationwide non-cooperation movement.	Approximately 91 people were reported killed; major highways were blocked, internet access was again shut down, and an indefinite nationwide curfew was imposed.	Such widespread disruption could substantially impair health-service access, field operations, surveillance, logistics and communication during a critical period of institutional instability.
5-Aug-24	Prime Minister Sheikh Hasina left Bangladesh; an interim government was subsequently announced.	The country entered a major political and administrative transition.	Leadership transition and institutional reorganization may have affected continuity of program governance, accountability and strategic oversight of national health priorities.
August–December 2024	Post-uprising transition and institutional reorganization.	Public institutions operated during a period of major political transition following the uprising.	Potential interruption or reprioritization of routine program management, monitoring and strategic planning.
2025	Prolonged interim-government period with continued political contestation, administrative disruption and public-sector unrest.	Political and administrative priorities remained focused on institutional reform and transition toward elections. Several secretary, assistant Sec. and senior government officer suspended and replaced.	Several EPI’s and senior official of Bangladesh Health Ministry were suspended or moved from Bangladesh create a gap in leadership level. Prolonged institutional transition may have competed with sustained attention to routine immunization performance, surveillance signals and corrective program action.
September 2025	Interim government announced open tendering for measles vaccine	in September 2025, the interim government led by Prof Dr. Muhammad Yunus stopped vaccine procurement through UNICEF and shifted to open tendering. UNICEF strongly opposed the change.	These initiatives severely delayed procurement timelines, inducing nationwide vaccine stockouts and consequently accelerating the accumulation of zero-dose children
January to March 2026	Measles resurgence in Bangladesh began	Bangladesh experienced a marked increase in measles transmission beginning in January 2026, with rapid nationwide expansion becoming evident by March 2026.	Thousands of patients (mostly under five age) were infected and hundreds of infected people died till than
